# Does Active Design Influence Activity, Sitting, Wellbeing and Productivity in the Workplace? A Systematic Review

**DOI:** 10.3390/ijerph17249228

**Published:** 2020-12-10

**Authors:** Lina Engelen

**Affiliations:** 1 Active Spaces, Thirroul, NSW 2515, Australia; lina.engelen@sydney.edu.au; 2 Sydney School of Public Health, The University of Sydney, Sydney, NSW 2006, Australia

**Keywords:** active design, office, physical activity, review, health

## Abstract

Active design is an emerging concept to incorporate physical activity into daily life through thoughtful design, and is often implemented in new building designs. It is, however, not known what evidence base there is to support the claims. Through this systematic review, the current evidence for active design was investigated. Seven databases were searched. A range of search terms relating to active design, physical activity, sitting, performance and wellbeing were used. After title and abstract screening of 1174 papers and full-text screening, 17 were selected for inclusion. The papers provided promising evidence of active design aiding a reduction in sitting and increase in standing time. Limited evidence was found for physical activity; a few studies reported an increase in step counts. Musculoskeletal effects were investigated in few studies, but there is some evidence of benefits to lower back pain. There was consistent evidence for better light and air quality, but no evidence for other features of the workplace environment. No conclusive evidence was found on associations between active design features and work performance. There is hence some evidence to support the benefit of active design on physical health; however, the dearth and heterogeneity of the study designs, measures and findings warrant further research.

## 1. Introduction

Physical activity has great proven benefits to physical and mental health. Conversely, physical inactivity has been recognised as a significant risk factor for obesity, chronic disease and premature death [1,2]. A recent economic analysis found the annual estimated cost from physical inactivity to health care systems worldwide was INT$53.8 billion [3].

However, excessive sitting has also emerged as a related risk factor for ill health and mortality [4,5]. Both physical inactivity and sedentary behaviour are problems exacerbated by modern work practices and workplaces, driven by rapid technological change over the last 50 years [6,7]. The World Health Organization (WHO) recognises the workplace as a key setting for interventions to improve physical activity levels, and advocates for changes in the built environment that support healthier lifestyles and wellbeing [8].

Workplace health promotion activities targeting physical activity have typically focused on work-based exercise and wellness programs and ergonomic furniture [9], such as sit–stand desks; however, an emerging concept relating to manipulation of the indoor built environment to encourage activity, known as Active Design [10], has recently gained traction. With many people in developed nations spending up to 90% of their day inside buildings [11], active design strategies that facilitate and provide opportunities for activity, particularly incidental movement integrated into usual daily work practices, have recently been incorporated into public health and urban planning guidelines in several countries [11,12,13,14]. The guidelines contain a set of recommendations around stair, lobby and elevator design, as well as for internal walking routes, strategic placement of amenities within office buildings and provision of facilities such as bicycle storage, locker and shower rooms, to encourage activity [11,13].

The concept of active design is a holistic approach that incorporates the structural design elements necessary for promoting physical activity and wellbeing, as well as the policies, workplace cultures, work styles and personal factors that promote these, in line with ecological models [15,16]. The general principle of ecological models of behaviour is that the environment facilitates the range of behaviour by promoting and sometimes demanding certain actions and discouraging or prohibiting other behaviours [17].

Active design hence works on the basis that behaviour can be influenced at an individual and population level by altering the environments within which people make choices (choice architecture). Choice architecture can be broadly defined as the organisation or presentation of options to help people make decisions, and thus may accomplish two things: it may make it easier for the user/building occupant to navigate complex choices, but it may also influence the choices they make. It is proposed that interventions, such as thoughtful design, provision and placement of stairs, kitchens and end of trip facilities typically require little conscious engagement on the part of the individual to realise their intended effects, mainly working via automatic or non-conscious psychological processes [18,19,20].

Active design is a relatively new concept and it is not clear how much is known about its impact on health- and work performance-related outcomes. The implementation of active design is in many cases a large investment, but can also have the potential to be applied to many occupants and visitors. As an emerging field with possible implications for public health, efforts must be made to continue to support active design with scientific evidence, such that a strong case can be made to governments and the private sector to broaden its implementation.

In order to chart the state of evidence for active design on healthy behaviour, wellbeing and work performance in the workplace, I performed a systematic review, presented in the following sections on this paper.

## 2. Materials and Methods

### 2.1. Search Strategy

A rapid literature review was conducted in September 2016 to examine the feasibility of the research question: “Can active building design support increased physical activity and reduced sitting at work, and how does it impact on worker wellbeing and productivity?” A small evidence base was found to support suggestions that active design could support less sitting and more movement in offices, with uncertain impacts on wellbeing and productivity. A comprehensive search strategy was carried out in December 2018 for the purposes of this systematic review. Systematic review registration number: PROSPERO CRD42016048165.

The author searched Scopus, Pubmed, CINAHL, Business Source Ultimate, ProQuest Central, PsycInfo, Web of Science and Cochrane Library. This set of databases was selected to ensure a wide search across disciplines. The search results were collated into an online systematic review platform, Covidence (www.covidence.org), in which title and abstract screening, followed by full-text screening by two reviewers were conducted.

**Figure 1:**
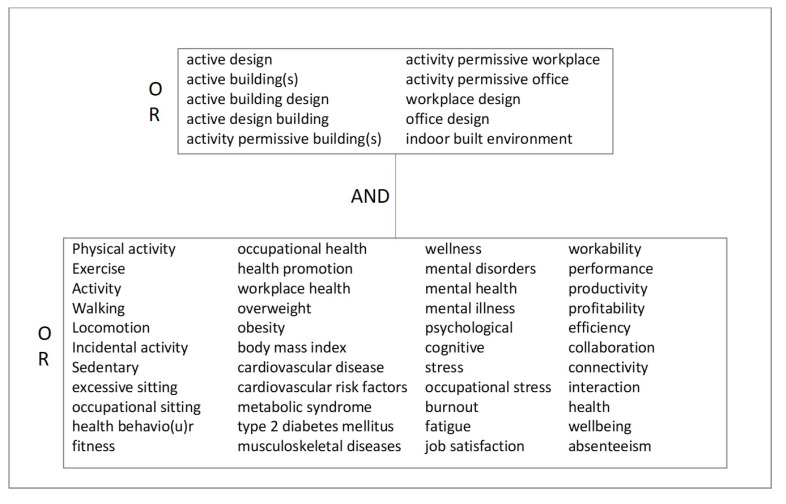
Search terms used in this systematic review.

Search terms used in the systematic review can be found in Figure 1. All these terms were used separated by the relevant Boolean operators, as stated in Figure 1. The same search strategy was used for all the databases.

### 2.2. Inclusions and Exclusions

Inclusion criteria for the studies were human studies (children or adults); English language papers; intervention or exposure involving the built indoor environment; intervention or exposure in a workplace or educational building; outcome(s) involving perceived or actual physical activity, movement, sitting, health, well-being, productivity or other factors relating to work performance; all study types (including conference papers, case studies and commentaries); and full-text paper available. Exclusion criteria were non-human subjects; not in English; intervention or exposure in residential buildings only; intervention or exposure involving only behavioural elements (e.g., workplace exercise programs, point-of-decision stair prompts, music in stairwells); intervention or exposure involving only ergonomic furniture (e.g., sit-stand desks, treadmill desks); intervention or exposure involving only the outdoor built environment; intervention or exposure involving only open-plan office design, activity-based working design or green design, without genuine active design elements; papers with no documented intervention or exposure; papers with no documented outcomes; and full-text paper not available.

### 2.3. Screening

**Figure 2:**
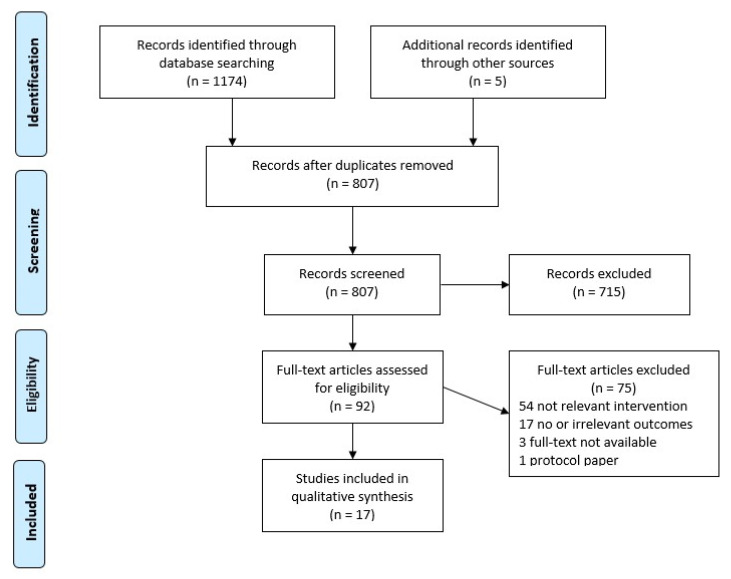
A flowchart of the database searches, screening and selection of articles according to the PRISMA statement (www.prisma-statement.org).

Figure 2 shows a flowchart of the database searches, screening and selection of articles according to the PRISMA statement (www.prisma-statement.org). A total of 1174 articles were added to Covidence for title/abstract screening, of which 1087 were deemed irrelevant; 5 additional papers were found through citation review, leaving 92 articles for full-text screen. Of these 92 articles, 75 articles were excluded at full-text screen by two independent reviewers, due to the full-text not being available (3 articles); irrelevant intervention (54 articles); and no or irrelevant outcomes (17 articles); protocol paper (1 article)—leaving 17 for data extraction.

Data extraction included summarising the study design, population, intervention(s) or exposure(s), outcome(s), exposure and outcome measures, overall results and practical implications. These results were categorized by the outcome measured and are presented descriptively below.

Quality assessment was done using a checklist developed specifically for this review, with elements taken from the National Heart, Lung and Blood Institute (NIH) Quality Assessment Tools for Before–After (Pre–Post) Studies [21] and Observational Cohort and Cross-Sectional Studies [22] as these were the predominant study types in the review. Quality items included clarity of research question; clarity of study population; acknowledgement and/or justification of sample size and participation rate; clarity of inclusion and exclusion criteria for participants; use of a comparator group; clarity of intervention/exposure descriptions; validity and reliability of exposure measures; clarity of outcome descriptions; validity and reliability of outcome measures; timeframe sufficiency to see an effect (if relevant); whether quantitative/statistical data analysis was attempted; acknowledgement of potential confounding factors; whether ethics approval was obtained; and generalisability.

## 3. Results

**Figure 3:**
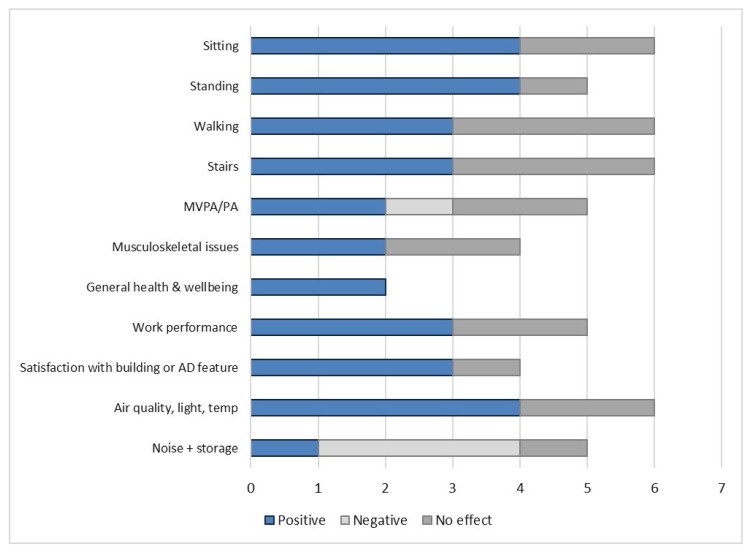
Summary of the number of studies that found a positive, negative or no effect on an outcome in the active design building. Sample size or the quality of the study have not been taken into consideration.

Seventeen [17] studies met our criteria for a study investigating how an active building design can support increased physical activity and reduced sitting in office workers, and how this can impact on worker wellbeing and productivity, and were included for data extraction in this review. Results are summarised in Table 1 and Figure 3. Full extraction information, including quality assessment, can be found in the online Supplementary Materials.

### 3.1. Types of Studies

Seven pre–post studies were found [23,24,25,26,27,28,39], investigating a range of outcomes after moving into new buildings incorporating active design elements. Four pre–post studies used accelerometer data to provide objective movement data [24,28,39]. Jancey et al. (2016) evaluated changes in activity among 42 adult office workers moving from a 1970s building to an “activity-permissive” building [39]. Engelen et al. studied 62 and 21 university staff moving from several older buildings into two new active design buildings [25,26] and measured self-reported movement, satisfaction and work performance variables with online surveys and focus groups 1 month prior to and 5 months after moving. Gorman et al. (2013) investigated movement and health-related outcomes in 24 adult physical activity researchers 1 week before and 4 months after moving into a new active design building [28]. Eyler [27] compared physical activity of participants moving and not moving from existing university building into a new building with active design elements, with a control group working in a different faculty. Creagh et al. [24] used mixed methods to study 42 adult office workers at a quasi-governmental peak body organisation before moving from a 1980s building and 3 months after moving into a purpose-built 5-star Green Star Building. Brown et al. (2010) [23] conducted a pre–post study of over one hundred workers moving to a new office with in-house fitness facilities with anonymous online surveys conducted 6 months pre- and 5 months post-move.

Seven cross-sectional analytic studies employing mixed method approaches were found, with four investigating the impacts of stair and elevator design [30,33,34,36], two investigating the influence of spatial and layout features on collaboration [31,32] and one exploring the impact of office layout and design on movement [35]. McGann et al. (2015) recruited 99 university staff across three buildings to wear accelerometers for a working week [33]. Bassett et al. (2013) used student observers to count the number of people taking elevators and stairs inside three university buildings [30]. Ruff et al. (2014) analysed self-reported stair use data from an online survey of 1348 employees in 14 office buildings [36] and Nicoll et al. (2009) [34] used self-reported stair-use measures and objective data from handrail sensors and card readers, over a 24-week period. Dell (2012) used a variety of qualitative and quantitative methods to investigate the impact of layout on collaboration of scientists in two university research buildings [31]. Hua et al. (2011) linked spatial analysis measures with self-reported perceptions of collaboration and distraction among 308 office workers in 11 buildings to produce a predictive model [32]. Rassia et al. (2011) studied 423 office workers in six workplaces and produced a mathematical model to predict activity based on spatial and layout variables [35].

Three qualitative studies were included, with two investigating employee attitudes toward sitting reduction strategies, including elements of active design [37,38], and one pilot study evaluating facilitators of and barriers to movement [29]. Hadgraft et al. (2016) conducted semi-structured interviews with 20 employees and managers from a range of industries [38], while Gilson et al. (2011) performed focus groups [37]. McGann et al. (2014) used ethnographic fieldwork methods to study the movement patterns of 90 government employees [29].

### 3.2. Outcomes

#### 3.2.1. Sitting and Standing

There is evidence for beneficial effects on standing and sitting, with four of the studies reporting beneficial effects on standing time (increase) [25,26,28,39] and three studies [25,26,39] found beneficial effects on sitting time (decrease), as measured by self-report or objective device. Engelen et al. [25] found a statistically significant increase in standing time and a decrease in self-reported sitting time in the new building, corresponding to an estimated 2.5-h reduction in total weekly sitting. In a larger study, Engelen [26] found that sitting as a proportion of the workday decreased from 83% down to 67% in the new building, with standing as a proportion of the workday increasing from 9% to 21%. This equated to an average of 1.2 h per day less sitting. Jancey et al. (2016) [39] found statistically significant reductions in sitting (85% to 80%), and standing as a proportion of the work day increased from 11% to 17% four months after moving. Gorman et al. (2013) [28] found a statistically significant increase in standing time (78 min per workday pre-move and 97 min post-move) after moving to a new building with active design features, and a corresponding reduction in sitting time (346 to 344 min per workday), which, however, only approached statistical significance. Conversely, Eyler [27] did not find any significant changes in sitting between the pre and post measures.

#### 3.2.2. Physical Activity

From this review there is inconclusive evidence that active design buildings are related to higher overall physical activity. Two studies by Engelen et al. [25,26] used self-report measures and neither of them found any changes in time spent walking or in moderate to vigorous physical activity (MVPA), nor in stair use. Similarly, Jancey et al. [39] found no effects of moving to an active design building on participants’ stair use or MVPA during work time, but they did find a significant increase in step counts four months after the move. However, this positive effect on step counts was not found in the study conducted by Gorman et al. [28]. Although, Eyler et al. [27] reported an increase in energy expenditure and step counts from pre to post in those who moved to a new building; similar results were found in both the non-movers and the control group, indicating that influences other than the building per se were at play.

In contrast, findings from one cross-sectional study, in which 99 university staff across three buildings wore accelerometers for a working week, indicates the beneficial effects of active design buildings on occupants’ MVPA and step counts [33], although these results were not statistically examined. Workers in the newest building, with attractive, open and naturally-lit staircases with outside views, spent more time engaged in moderate to vigorous activity than those in the older two buildings, which both had enclosed and artificially lit fire stairs. Workers in the newest building also recorded the highest mean step count.

Two cross-sectional studies that focused on stair use found increased stair use when the stair wells were accessible and well lit [30,36]. Bassett et al. (2013) [30] used student observers to count the number of people taking elevators and stairs inside three university buildings, and found stair use far outweighed elevator use in the two buildings with centrally located, well-lit, attractive and easily visible staircases, as opposed to the building with a central elevator bank and concealed, enclosed fire stairs. Workers were more likely to use stairs that were naturally lit and that were more easily visible from the entrance lobby [36].

Nicoll et al. (2009) [34] also reported on the effect on the attractiveness of the stairs and function of the lifts. They used self-reported stair-use measures and objective data from handrail sensors and card readers over a 24-week period, and found that an open staircase adjacent to a “skip–stop” elevator that stopped every 3rd floor was used 33 times more than an enclosed fire stair adjacent to a traditional elevator (in the same building) over a 24-week period.

A study using a variety of qualitative and quantitative methods to investigate the impact of layout on collaboration of scientists in two university research buildings, found that researchers were more likely to move if spaces were well-integrated and easily visible via thoughtfully designed corridors [31]. Similarly, Rassia et al. (2011) [35] studied 423 office workers in six workplaces and produced a mathematical model to predict activity based on spatial and layout variables and found that movement patterns inside office spaces depended on the number and size of windows, having different office destinations and accessible stairways.

One study conducted semi-structured interviews with 20 employees and managers from a range of industries [38], and thematic analysis found that centralised facility hubs (print/copy stations and bin rooms) did not always encourage more frequent walking, with workers reportedly saving up rubbish or print/copy jobs and limiting trips to once a day or once every few days. Conversely, in a focus group-based study by Gilson et al. (2011) [37], workers expressed interest in walking further to reach centralised facility hubs, including toilets located on other floors. Inaccessible, enclosed stairwells limited stair use, and in both studies the workplace culture was described as a barrier to movement. McGann et al. (2014) [29] used ethnographic fieldwork methods to study the movement patterns of 90 government employees and found that workers moved primarily to access food/drink, to be with others or to complete paperwork tasks, such as printing/copying. Barriers to activity included aggressive signage on closed fire stair doors, inappropriate footwear and safety concerns, and lack of accessible and easy-to-navigate internal circulation routes.

#### 3.2.3. Musculoskeletal Effects

Only two studies, both by Engelen and colleagues [25,26], assessed impacts on musculoskeletal issues. These two pre–post studies were consistent in their results; moving to a new building with active design features decreased lower back pain and had no impacts on pain or discomfort at other body sites (neck or shoulders, hands or arms, leg or joints).

#### 3.2.4. Work Performance and Health

There is some evidence that aspects of work performance and health were positively related with active design buildings. Data suggests positive effects on finding work motivating and looking forward to going to work [25,26], but there were no effects on perceptions of being able to concentrate at work, efficiency, quality of work, amount of work or preference to work at home. Jancey et al. (2016) [39] assessed perceptions of work performance and job satisfaction, and found no changes in these variables after moving to the new building. Conversely, perceptions of general productivity were observed to improve post-move by Brown et al. [23], but this was not tested for statistical significance. Creagh and colleagues [24] found that participants in the new building had higher levels of self-reported feelings of energy and overall health in the new building. Engelen (2016) [25] found that the participants were less tired when leaving for work in the Active Design environment; however, this effect was not observed in Engelen et al. (2017) [26].

#### 3.2.5. Perceptions of the Workplace Environment

Four studies, three pre–post studies [24,25,26] and one cross-sectional study [23], investigated the overall satisfaction with the building, as well as a range of indoor environmental quality aspects, such as noise, air quality, light and temperature, in addition to the perception of sufficient storage. Creagh et al. [24] reported a higher percentage of participants satisfied with the overall building in the new building in comparison to the old building (77.4% and 51.6%), although no statistics were reported. Engelen et al. [19] found no changes in perception of noise, while Engelen et al. [25] found moving to a new building had a negative impact on perceptions of noise. Brown et al. [25] reported improved perceptions of noise after moving.

Improved perceptions of air quality and light in the new building were fairly consistent across the studies. Engelen et al. (2017) [26] found improved perceptions of temperature, while another pre–post study [25] found no changes in the perceptions of temperature, as was the case in Brown et al.’s study [23]; although, the latter study results were not tested statistically. Two studies [25,26] reported negative perceptions of storage in the new building compared to the previous building. In contrast, Brown et al. found positive perceptions of storage in the active design building [23].

## 4. Discussion

In this systematic review on the evidence of active design strategies’ influence on health and work performance measures in the workplace, 17 papers were identified that met the inclusion criteria. This relatively low number is not surprising, given the emerging nature of the topic. There is good evidence for sitting and standing. Consistent evidence that active design strategies increase standing and reduce sitting during the workday was found throughout the studies. Given the emphasis on stair design in these buildings, it is surprising that the pre–post studies did not report increased stair use; however, the cross-sectional studies generally found positive associations between improved stair design and stair use.

The evidence for increased walking or stepping in active design buildings was inconclusive in the present review, with the one study that found evidence for increased walking [33] attributing this to more incidental movement to and from centralised facility hubs; however, the other three studies of buildings with similar centralised hubs failed to find significant increases in walking [25,26,28]. These findings suggest that effort needs to be made to not only design for more walking opportunities, but also to support more movement throughout the office, for example, by stimulating usage of meeting rooms and kitchen facilities in different parts of the workplace, or on different floors. There was a consistent effect on musculoskeletal issues, particularly a beneficial effect on low back pain; however, only two studies examined this. Previous studies have also suggested a relationship between activity-permissive offices, less sitting and less lower back pain [40,41].

The research revealed paradoxical impacts of active design strategies, such as an unexpected increase in workers identifying as disabled when moving to a building with a skip–stop elevator [34], and the deliberate avoidance of walking to centralised facility hubs, with workers saving up rubbish and print/copy jobs [38]; this despite findings in several studies to support centralised facility placement as a useful active design strategy to increase incidental movement. Importantly, qualitative data provided evidence for a need to combine structural active design strategies with approaches aiming to alter workplace culture and social norms, such that workers feel movement is acceptable.

Only a small number of studies reported productivity or motivational outcomes; some positive effects on finding work motivating and looking forward to going to work were found, but not much evidence for effects on work performance. A previous study by Dole and Schroeder (2001) stated that, in the workplace, it is often assumed that employees who are more satisfied with the physical environment are more likely to produce better work outcomes [42]. This was corroborated in two previous studies looking at the effect of office design on work performance [43,44]. Although the studies we reviewed did not report this relationship between active design and work performance, it is possible that there could be an indirect link between them. This discrepancy is an avenue for future research.

There was consistent evidence for perceived better light and air quality in active design buildings, but no evidence for other features. This outcome is likely more related to the buildings being relatively new and fresh, rather than the active design strategies *per se* [45]. However, there is ample evidence that indoor environment quality factors, such as light, noise and air quality, has large effects on wellbeing and performance [46]; hence, effort should be maintained to optimise these.

The built structures alone may not change behaviours of its users [47,48]. To be effective, we need a combination of disciplines to apply principles of active design across all levels, from the physical structure of the building to the personal, social, cultural and political environment, in line with ecological models.

As some managers identified in one qualitative study [38]; it is necessary to underpin the push for workplace activity strategies (including active design), with solid evidence for efficacy relating to health outcomes, as well as for cost-effectiveness relating to productivity and work performance outcomes, such that decision-makers are swayed to support changes in the face of limited resources and competing demands. Active design depends greatly upon the willingness of political, academic and business leaders to hold long-term visions and advocate for multisectoral collaboration, supported by policy and regulatory measures.

### 4.1. Strengths and Limitations

This systematic review highlights the multitude of disciplines that study active design for various reasons. The diversity of disciplines conducting and reporting on active design settings and its effects is a strength in this field, but this also raises a number of issues that make it hard to draw solid conclusions based on the studies included for review. One challenge is the variation in research methods due to the nature of the multidisciplinary backgrounds of researchers investigating active design. While more rigorous scientific methods including pre–post-studies with comparison groups may generate usable evidence, such studies are often limited in scale. In comparison, post-only studies involving large samples, common to the architecture and corporate property fields, may provide more generalisable data, but with weaker evidence of causality (i.e., that the changes in observations are due to the active design environment). Other studies just assess the use and implementation of active design, and provide process measures, but not evidence of actual impacts. For these reasons, cross-disciplinary reviews have challenges in reaching clear conclusions, and this is a limitation of the current review.

While some studies used objective outcome measures, such as accelerometers and handrail sensors, there was a general reliance upon subjective self-reported outcome measures, which may have reduced validity of the results and introduced bias. Generalisability may be limited by the high proportion of studies conducted in academic or research buildings, and an absence of studies involving blue-collar workers.

A limitation of this review was that the search methods may not have uncovered the full evidence base, due to exclusion of non-English studies, review of a finite number of databases and a decision to not actively contact researchers to request unpublished papers.

### 4.2. Recommendations for Future Research

Based on the outcomes of this review, it is evident that a number of gaps in the literature around active design exist, and especially the lack of objective measures and of evidence on health and work performance effects. Going forward, future studies should attempt to measure a range of outcomes across a variety of settings. To progress with strengthening the evidence base, further emphasis should be placed on using consistent and appropriate evidence-generating research methods and designs across disciplines, to ensure comparability. Health outcomes need to be more specific than “overall health”, to tease out if the effects are physical, mental or whether they are a reflection of the organisation.

## 5. Conclusions

Active Design is a recent concept and is often incorporated in many new office building designs. It has some proven and a range of other potential benefits for health, wellbeing and productivity. The evidence base for active design is limited, although at this stage the evidence to support the benefit of active design on sitting, standing, step count and musculoskeletal issues seems quite convincing. The range of studies is often characterised by non-comparable measures, but this is reflective of real-world settings. The implementation of active design is often accompanied by significant investments, both in terms of time and planning as well as finance. It is therefore important to support claims of efficacy with further evidence to ensure the best investments for public health are made. Future research to build the evidence base of the effects of active design on health, perceptions and productivity is warranted.

## Figures and Tables

**Table 1:** Summary information of data extraction divided by type of study design.

Study, Country	N	Exposure(s)/Intervention(s) and Descriptions	Major Findings
**Pre–Post Studies**			
Brown et al. (2010), Canada [23]	145	Office workers moving from an older building to new “green” interaction-focused building with health and fitness facilities. Observational descriptive data on old and new buildings provided.	Workers perceived their health to have improved post-move, and cited provision of a workplace gym and sense of increased emphasis on wellbeing as reasons. There were reported improvements in comfort and productivity, mainly related to better environmental conditions.
Creagh et al. (2017), Australia [24]	42	Adult office workers at quasi-governmental peak body organisation moving from a 1980s building to a purpose-built 5-star Green Star Building. The organisation’s wellness committee had been part of the design process.	Increase in self-reported feeling of energy, overall health and satisfaction with the overall building in the new building. Small reduction in accelerometer-measured sedentary time (84.9–79.7%) and corresponding increase in light physical activity (11.2–17.0%) in the new building. No change in MVPA was observed. Discussion of environmental factors that support and inhibit physical activity.
Engelen et al. (2016), Australia [25]	21	Adult university staff moving from several older buildings into one new building, designed using Active Design principles. Exposures assessed via building audit.	Workers sat less and stood more (average of 1.2 h per day less sitting), but there were no changes in walking or stair climbing. Workers reported less back pain, but productivity was unchanged.
Engelen et al. (2017), Australia [26]	62	Adult university staff moving from several older buildings into one new building, designed using Active Design principles, with longer and attractive walking routes to centralised facilities, naturally-lit open central staircase and bicycle storage and showers. Exposures assessed via building audit (observational and measurement data).	Workers sat less and stood more in the new building (approximately 2.5 h per week less sitting), but there were no changes in walking or stair climbing. Workers reported less back pain, more motivation and were more satisfied with their environment and connectivity. Sleep and productivity were unchanged.
Eyler et al. (2018), United States [27]	166; 89	Adult university staff moving or not moving from existing building into new building and control participants in a different building where no change took place. The new building had large, open and centrally located stair wells; sit/stand desks; end-of-trip facilities; and centralised printing and rubbish facilities.	Increase in self-reported sit/stand workstations (27.9–65.8%); change facilities (25–50.4%) and support for PA (33.9–47.0%). No change was seen in self-reported PA. Although the objective measures showed increases in energy expenditure and step counts from pre to post in the Movers, significant increases were also measured in the Non-movers and the control participants.
Gorman et al. (2013), Canada [28]	24	Adult workers in a physical activity academic research centre, moving from an older building into a new purpose-built building designed to support movement, including glass staircases, centralised and vertically integrated facilities. Observational descriptive data on old and new buildings provided.	Workers spent more time standing and less time sitting in the new building, but there were no significant changes in walking, body composition, health, work performance or satisfaction measures.
Jancey et al. (2016), Australia [29]	42	Adult office workers moving from 1970s building to new “activity-permissive” purpose-built building, with more accessible circulation routes, attractive central open staircase and further walking distances to centralised amenities, such as print room, toilets, kitchen. Exposures assessed via building audit.	Workers spent less daily work time sitting down and more time standing up in the new building, and, on average, took more steps each day. Time spent doing moderate or vigorous activity did not change post move, with no changes in stair use. Average duration of sedentary bouts actually increased.
**Cross-Sectional Analytic Mixed-Method Studies**			
Bassett et al. (2013), United States [30]		Employees and visitors to three buildings on a university campus, where buildings had different stair, elevator and lobby design characteristics. Observational descriptive data on building design provided.	Far greater proportions of people used stairs in the two buildings which had centrally located, attractive, well-lit and accessible staircases, compared with those in the building with central elevator bank and closed fire stairs located further from lobby entrance.
Dell et al. (2012), United States [31]		Researchers, laboratory technicians and students working in two university science buildings. Space Syntax Analysis used to quantitatively assess spatial features of each building in addition to qualitative descriptions of layout and spatial features.	Workers were more likely to move within their building if spaces were more integrated, particularly via well-designed corridors. Social interaction, which may be critical to collaboration, is supported by having access to shared break and laboratory equipment areas. Use of shared spaces is facilitated by placing them near integrated corridors to increase accessibility and visibility.
Hua et al. (2011), United States [32]	308	Office workers in 11 buildings (10 federal workplaces and 1 research building) engaging in their typical workplace activities over the study period. Five workstations and six floor plan variables were measured for spatial analysis.	Office floor-plan spatial characteristics are more important than workstation characteristics in predicting perceptions of support for collaboration among workers. Locating kitchens and print/copy areas in centralised hubs some distance away from workstations can reduce distractions and improve perceptions of both informal and planned collaboration.
McGann et al. (2015), Australia [33]	99	University staff working in one of three buildings, built in either the 1970s, 1980s or 1990s. Relevant features of newest building included open and well-lit staircases and centralised facilities, compared to fire-stairs in older buildings. Architectural audit provided exposure information.	Workers in the newest building (with more attractive and accessible stairs) spent more time engaged in moderate-vigorous activity during the workday than those in the two older buildings, presumed to be largely due to increased stair use.
Nicoll et al. (2009), United States [34]	299	Office workers in a 13-storey government building with one skip–stop elevator vertical circulation core (elevator stopping every 3rd floor for able-bodied workers, with adjacent open stairwell) and one traditional elevator circulation core with adjacent enclosed fire stairwell. Descriptive data regarding building design elements provided.	The open staircase adjacent to the skip-stop elevator was used 33 times more than the fire stairs adjacent to the traditional elevator. However, one quarter of building occupants were dissatisfied with the skip-stop system, and there was an unexpected rise in the number of people identifying as disabled upon relocating to the building with the skip-stop system. Interviews identified that installation of skip–stop systems required deviation from normal building codes and security issues may occur with an open continuous stairwell if one building houses multiple companies.
Rassia et al. (2011), United Kingdom [35]	423	Office workers in six offices, observed going about their typical daily tasks over a 2-week period. Indoor office layout data collected via observations and measurements.	Office workers frequently visit kitchens and print rooms. An increase in workstation-to-kitchen distance will not discourage kitchen use but distance will negatively influence trips to the print room. Trips to toilets, managers’ offices and meeting rooms occur at the same frequency irrespective of office layout. The presence of larger windows, allowing more natural light and outside views, at a given destination will encourage movement to that destination. Intra-office distances longer than 50 m, and stair travel beyond two flights are likely to discourage walking. Decreasing the speed of elevators and distance from workstation to stairs may increase stair use.
Ruff et al. (2014), United States [36]	1348	Employees from 14 office buildings in New York City. Exposure measures consisted of qualitative observational and quantitative measurement data on stairwell and lobby design spatial characteristics.	Office workers were more likely to use stairwells that were naturally lit and those that had greater visibility from the entrance lobby. Workers were less likely to use stairs when they worked on a higher floor or if the stairs were located further from the lobby entrance.
**Qualitative Studies**			
Gilson et al. (2011), Australia [37]	24	Government office workers from city and regional locations. Descriptive data on office environments obtained via focus groups.	Workers were open to ideas around centralising printers and bins in hubs located further away from workstations, and would be willing to walk further to get coffee or use the toilet, including accessing bathrooms located up or down stairs. Barriers to moving more in the office included inaccessible stairwells, concerns about productivity and loss of concentration, and workplace culture where being away from a desk may be seen in a negative light.
Hadgraft et al. (2016), Australia [38]	20	Employees and managers from a range of industries in office-based settings where formal sitting reduction strategies had not been employed. Descriptive data on office environments provided by authors and generated through semi-structured interviews.	Centralised facility hubs (printing/copying, rubbish bins, kitchens) did not always encourage more worker movement, and may have a paradoxical effect, whereby workers save up print/copy jobs to avoid walking regularly to the facility, and collect mounds of rubbish by their desks during the day, taking this to the bins room once a day or every few days. Managers identified need for strong business case to support sitting reduction strategies.
McGann et al. (2014), Australia [29]	90	Local government employees continuing their usual work in an older style office building. Spatial characteristics gathered by observation and architectural floor-plan review.	Workers move primarily to be with others, to access food or drink, and to complete paperwork tasks, such as printing and photocopying. Workplace culture was a facilitator of movement. Barriers to movement and stair-use include lack of easy-to-navigate circulation routes through the office, inappropriate footwear, safety concerns, physical barriers such as aggressive signage on closed fire stair doors.

## Supplementary Materials

The following are available online at https://www.mdpi.com/1660-4601/17/24/9228/s1. Table S1: Full extraction table.
